# Editorial: Prediction and explanation in biomedicine using network-based approaches

**DOI:** 10.3389/fgene.2022.967936

**Published:** 2022-09-02

**Authors:** Alessio Martino, Alessandro Giuliani

**Affiliations:** ^1^ Department of Business and Management, LUISS University, Rome, Italy; ^2^ Department of Environment and Health, Italian National Institute of Health, Rome, Italy

**Keywords:** network inference, explainable artificial intelligence, pattern recognition, biological network analysis, network modelling

The complex network paradigm occupies a twilight epistemological status in between data analysis and causal, content based, modelling of complex systems. This status is mirrored by the title of the topic “*Prediction and Explanation in Biomedicine using Network-Based Approaches*” putting together “prediction” (data analysis perspective) and “explanation” (causal modelling perspective). Scientific methodology is aware of the different, albeit related, status of the two perspectives since long time (Shmueli, 2010) and the actual emphasis of machine intelligence community on “explainability” revived the urgency of the issue (Ho et al., 2020).

As aptly stated by Nicosia and others (Nicosia et al., 2014):

“Networks are the fabric of complex systems”.

while, at the same time, being a very flexible data analysis tool inheriting from time-honoured multidimensional statistics the focus on correlation matrices (Gorban et al., 2022).

Biological systems are the most evident paradigm of complexity, and this is why it is much more productive to focus on the dynamics of their correlation structure with respect to an in-depth analysis of isolated features. In this Research Topic, this point is made evident by papers exploring correlation structures located at different organization layers: contacts between amino-acid residues of a protein molecule (Uversky and Giuliani), gene expression correlation (Tran et al.) and protein-protein interaction networks (Cesareni et al.; Wang et al.). In particular, Uversky and Giuliani review the most recent results in terms of hierarchical organization of complex biological systems, remarking the benefits of analyzing such systems in a multi-level fashion, hence going beyond the standard causative model where events originate at molecular level and then show up at the ‘top’ of the hierarchy (e.g., causing a particular disease). Causality is also the key in (Cesareni et al.), where the authors review how causality can help in shaping disease networks, shedding light on using also functional information alongside physical proximity (i.e., between interacting proteins) for a thoughtful modelling. In Tran et al., the authors question the suitability of MCF-7 cell line for *in vitro* breast cancer research. They use a network-based approach to compare two MCF-7 datasets against a human breast invasive ductal carcinoma dataset taken from The Cancer Genome Atlas (TGCA), showing how they have only minimal similarity in biological processes, hence concluding that using MCF-7 to study breast cancer can hide important gene targets. Finally, TGCA plays an important role also in (Wang et al.), where the authors use a network-based approach to find hub genes related to acute lymphoblastic leukemia.

It is important not to confuse the integration of data analysis and explanatory perspectives with the too-often repeated statement of the substantial irrelevance of the hypothesis-driven approach when in presence of massive amount of data (Mazzocchi, 2015); the situation is exactly the opposite: network paradigm asks for a strict integration between content related and methodological knowledge and the consequent need to overcome research overspecialization. It is not by chance that very interesting new perspectives in statistical mechanics generate from the analysis of biological network systems (Liu et al., 2022): along this line, in this Research Topic, we find papers devoted to theoretical/computational issues (Kuznetsov et al.; Nazarenko et al.) motivated by the solution of relevant biomedical problems. Specifically, Kuznetsov et al. use a variational autoencoder to generate a synthetic 1-cycle ECG which not only looks quite natural, but can also be generated starting from just 25 features automatically learned by the autoencoder. As instead, Nazarenko et al. show an interesting network-based approach based on parenclitic and synolytic networks to describe multidimensional data via a suitable graph that makes the data easier to inspect, visualize and analyze. Tests on synthetic and benchmark data corroborate the competitiveness of using parenclitic and synolytic networks against common machine learning approaches.

In this Research Topic, the application potential to biomedical practice of network-based approaches is explored in (Chen et al.; Jung et al.; Luo et al.; Thomas et al.), that give us the strong impression that network-based approaches are here to stay. In detail, Thomas et al. review how network biology can help in understanding inflammatory bowel disease by discussing different network modelling (notably, protein-protein interaction networks, metabolic networks, gene regulatory and co-expression networks), with some examples also on multi-layered networks. Chen et al. build a predictive model based on network analysis and circular miRNA to address recurrent implantation failure (RIF). Luo et al. also aim at characterizing RIF, but the authors exploit network-based approaches (protein-protein interaction and circRNA–miRNA–mRNA networks) to highlight four hub genes that may be involved in the development of RIF. Finally, Jung et al. briefly review computational models based on machine learning, network modelling and genome-scale metabolic models to characterize drug-resistant cancer cells.

After all, this is not surprising at all, “network biology” is nothing else than “biology as such” given any biological system derives its peculiar behaviour from the interaction of many different element players at different organization layers with no privileged causal layer of explanation (Noble et al., 2019). This is a bare truth (too often overlooked) since the initial definition of “Organism” in classical philosophy (Gotthelf and Lennox, 1987); what is new is the exciting possibility to use these concepts in the day-to-day practice of biomedical sciences by an immediate hands-on approach: we do hope the present Research Topic to transmit this excitement to the reader.
